# Development of an Ecological Momentary Assessment Study to Identify Real-Time Predictors of Physical Activity Among Older People With HIV: Protocol for a 2-Phase Mixed Methods Study

**DOI:** 10.2196/81238

**Published:** 2025-12-09

**Authors:** Dan Song, Lisa Hightow-Weidman, Tuzhen Xu, Yijiong Yang, Jing Wang

**Affiliations:** 1 Texas A&M University – Corpus Christi Corpus Christi, TX United States; 2 Florida State University Tallahassee, FL United States; 3 Prairie View A&M University Houston, TX United States

**Keywords:** HIV, physical activity, predictors, ecological momentary assessment, protocol

## Abstract

**Background:**

People with HIV are aging rapidly and face accelerated aging-related comorbidities, including cardiovascular diseases and cognitive impairment, due to prolonged HIV-associated inflammation. Physical activity (PA) is a well-established intervention to mitigate these risks; yet, most older people with HIV remain sedentary. Despite considerable efforts to understand PA determinants and design interventions for people with HIV, outcomes have been suboptimal.

**Objective:**

The overarching goal of this project is to use an ecological momentary assessment (EMA) approach to capture ecologically valid relationships between the personal experiences of older people with HIV and PA, as they engage in their normal daily activities.

**Methods:**

This study will adopt a 2-phase mixed methods research design. The first phase focuses on developing the EMA questionnaire through in-depth interviews with older people with HIV to explore the relevance of candidate real-time predictors of PA, identified using the capacity, opportunity, motivation—behavioral framework and literature review, to their daily experiences. These interviews will validate and refine the constructs for the EMA survey. In phase 2, a 2-week EMA study will collect data from 70 sedentary older people with HIV through smartphone surveys (3 times per day) and Fitbit-measured step counts and moderate to vigorous intensity PA minutes. Multilevel modeling will be used to examine how these factors predict daily PA levels.

**Results:**

This research project was funded in June 2024. To date, 13 eligible participants have completed the qualitative interviews. All participants agreed that the constructs of EMA survey are relevant to their PA experiences and acknowledged their time-varying nature. On the basis of the participants’ input, the EMA survey has been finalized.

**Conclusions:**

By advancing the understanding of real-time determinants of PA, this study addresses a critical gap in the literature and offers a foundation for designing just-in-time adaptive interventions that provide tailored, context-specific support to enhance PA engagement and promote healthy aging among older people with HIV.

**International Registered Report Identifier (IRRID):**

DERR1-10.2196/81238

## Introduction

### Background

This paper describes the rationale and design of an ecological momentary assessment (EMA) study to improve understanding of physical activity (PA) among older people with HIV. By capturing within-person, time-varying factors that influence the daily PA levels of older people with HIV, it is anticipated that the study will offer novel insights into predicting PA behavior among older people with HIV. The study findings are expected to lay the foundation for designing precision-based PA behavioral interventions tailored to participants’ individualized needs in real time, which will increase PA levels effectively and lead to improved health outcomes and reduced health care burdens for both older people with HIV and their caregivers. In doing so, it addresses health disparities in the context of healthy aging among older people with HIV.

According to the World Health Organization, approximately 39 million people worldwide were estimated to be living with HIV by the end of 2023; in the United States, the number was approximately 1.2 million [1]. Owing to the introduction of effective antiretroviral therapy, life expectancy in people with HIV has significantly improved, leading to HIV being increasingly considered a chronic disease [2]. In the United States, approximately 52% of people with HIV are aged >50 years [3], and this is projected to increase to 70% by 2030 [4]. The advancing age in people with HIV carries an increased risk for a wide range of comorbid conditions, such as cardiovascular diseases and cognitive impairment, due to prolonged HIV-associated inflammation, distress, and side effects of antiretroviral therapy [5,6]. Indeed, older people with HIV are 50% more likely to develop cardiovascular diseases [7] and 58% more likely to develop dementia [8], which often interferes with medication adherence [9] and is associated with a higher mortality rate [10]. PA, as an established lifestyle intervention, plays a crucial role in promoting overall well-being [11]. To achieve substantial health benefits, PA needs to be performed regularly and maintained across the life span [12]. However, less than 20% of older people with HIV engage in at least 150 minutes of moderate to vigorous intensity PA (MVPA) per week [13], as recommended by public health guidelines [14]. Given the growing number and health burden of older people with HIV, there is a pressing need for a deeper understanding of the predictors of PA levels among this group.

Historically, methods for measuring PA have been limited by the lack of technologies capable of capturing behavior in real time. Most research has relied on cross-sectional study designs to identify correlates of PA participation in people with HIV. For example, a recent quantitative systematic review of 45 cross-sectional and cohort studies highlighted key barriers to PA among people with HIV [15], including older age, lower educational attainment, decreased CD4^+^ T-cell count, exposure to antiviral therapy, body pain, and depression. While these studies have provided valuable insights, they primarily focus on between-person factors (interindividual differences) rather than within-person factors, such as daily experiences or situations, that may influence PA levels. Consequently, little is known about how time-varying, context-specific factors impact daily PA levels among older people with HIV.

Advances in mobile and sensor technologies offer promising solutions to these methodological limitations. These technologies enable real-time data collection strategies, such as EMA, which is particularly well suited for examining time- and context-dependent fluctuations in PA determinants [16]. EMA involves the use of smartphones to collect real-time self-reports of behaviors, contexts, emotional states, beliefs, attitudes, and perceptions in naturalistic settings [17]. By capturing data proximal to the time and place of behavior, EMA reduces recall errors and biases, enhancing ecological validity [18]. Unlike traditional cross-sectional or retrospective methods, EMA is uniquely equipped to study phenomena that vary over time, offering a dynamic perspective on PA behavior and its correlates [19]. This approach provides opportunities to test and refine explanatory models, enabling researchers to gain deeper insights into the mechanisms underlying PA behaviors.

The application of EMA in PA research is expanding rapidly. This time-intensive approach has demonstrated the ability to generate novel insights into the real-time determinants of PA behavior. However, to our knowledge, no studies have used EMA to investigate real-time predictors of PA among older people with HIV. This represents a critical gap in the literature, as understanding these predictors is essential for developing tailored, real-time behavioral interventions to promote PA in this population. Older people with HIV face unique challenges in maintaining sustained PA engagement and experience significant health disparities in healthy aging outcomes.

This study aims to address this gap using EMA to explore real-time predictors of PA among older people with HIV. These insights are essential for the development of just-in-time adaptive interventions (JITAIs), which can deliver tailored, context-specific support to promote sustained PA engagement and improve health outcomes in this vulnerable population.

### Theoretical Framework

Among various syntheses of different theories, the capability, opportunity, motivation, and behavior (COM-B) model, rooted in evidence and derived from 19 frameworks of behavior change theories, serves as a comprehensive framework for understanding behavior change [20]. The COM-B framework consists of 3 constructs: capability, opportunity, and motivation.

Capability within the COM-B model encompasses a person’s knowledge, cognitive abilities, and physical strengths necessary to perform a health behavior [20]. Among these factors, physical strengths, such as fatigue and pain, are inherently time varying. Fatigue and pain are among the most prevalent symptoms experienced by older people with HIV. Research indicates that approximately 55% to 65% of older people with HIV report fatigue [21], whereas chronic pain affects an estimated 30% to 80% of this population [22,23]. Both quantitative and qualitative studies consistently identify fatigue and pain as significant barriers to PA engagement among older people with HIV [15,24]. However, the relationships between these physical experiences and PA are seldom examined using within-person methods. Differentiating within-person versus between-person associations between the physical experiences of older people with HIV and their PA levels would provide valuable insights into whether and how these experiences influence PA daily. Such insights could inform tailored interventions aimed at mitigating these barriers to promote sustained PA engagement in this population.

Opportunity refers to external factors that either facilitate or hinder PA participation [20]. These include environmental factors (eg, access to safe and convenient exercise facilities), social factors (eg, support from family or friends), and economic factors (eg, availability of affordable sports equipment). While environmental and economic factors are relatively stable and will not be explored in this study, the dynamic nature of social interaction suggests it can fluctuate throughout the day, influenced by factors such as availability of social support. Previous studies have highlighted that social support is one of the strongest facilitators of PA engagement [24]. Understanding these fluctuations allows a more nuanced view of how social interactions affect PA engagement in real time among older people with HIV.

Motivation, within the COM-B framework, is categorized into 2 primary types: reflective and automated [20]. Reflective motivation encompasses conscious and deliberate processes that shape behavior, whereas automated motivation is primarily driven by emotions. In this study, we examine intention and mood as proxies for reflective and automated motivation, respectively. Both factors are dynamic and subject to contextual fluctuations over time. While substantial research has demonstrated that PA engagement is negatively associated with concurrent negative affect [25], there has been limited empirical investigation into whether negative or positive affect prospectively predicts PA. Gaining a deeper understanding of the relationship between mood states and subsequent PA among older people with HIV could provide valuable insights into the emotional predictors of PA in this vulnerable population.

In summary, the COM-B framework is used to identify candidate real-time determinants of PA levels among older people with HIV. Informed by the COM-B framework and a literature review, 5 candidate real-time factors are proposed: pain, fatigue, mood, intention, and social support. The relevance of these determinants to the daily PA experiences of older people with HIV will be examined through qualitative interviews. Subsequently, a final list of real-time determinants of PA will be developed and tested in an EMA study.

### Aims

The overall aim of this study is to determine the real-time predictors of PA levels among older people with HIV. The following 2 specific aims are proposed:

1. To develop the EMA survey based on the theory, literature review, and inputs of older people with HIV. To achieve this aim, initially, the team will use the COM-B framework to draft a list of potential real-time predictors of PA based on existing literature. Following this, in-depth qualitative interviews will be conducted with older people with HIV to validate, expand upon, and identify additional predictors of daily variations in PA level within this population.
2. To implement the EMA study to determine the role of the candidate real-time predictors of PA level (identified in aim1) in predicting variations in PA levels (step counts and MVPA minutes assessed by Fitbit [Fitbit Corporation]) among older people with HIV. This aim will use a 2-week longitudinal design that uses bursts of mobile-based surveys (3 times per day) with older people with HIV (n=70). Multilevel models that address the nested structure of EMA data will be used to evaluate the study aims.

## Methods

### Overview of Research Approach

**Figure 1:**
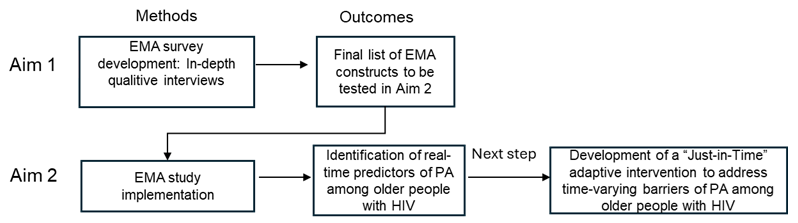
Overview of the research approach for the whole study. EMA: ecological momentary assessment; PA: physical activity.

Figure 1 depicts the overview of the research approach for the entire study. This study will adopt a mixed methods research design. The first phase focuses on developing the EMA questionnaire through in-depth interviews with older people with HIV to explore the relevance of candidate real-time predictors of PA, identified using the COM-B model and literature review, to their daily experiences. These interviews will validate and refine the constructs for the EMA survey. In the second phase, the finalized survey will be implemented in a 2-week EMA study to examine the role of these predictors in influencing variations in PA levels, measured through Fitbit step counts and MVPA minutes, among older sedentary people with HIV. Findings from this study will lay the foundation for designing JITAIs to address time-varying factors that impact daily PA levels in this population.

### Methods for Aim 1: Development of the EMA Survey

#### Research Design

This phase of the study involves conducting in-depth structured interviews to explore the perspectives of older people with HIV on the relevance of items derived from the literature review and COM-B theory to their own PA behavior. We plan to engage approximately 10 to 15 participants. Additionally, the interviews will solicit input on constructs or items that may have been overlooked during the initial review. These interviews will validate the relevance of identified predictors and offer critical insights to refine and expand the constructs for the EMA study outlined in aim 2. This iterative approach [26] will ensure that the EMA survey comprehensively captures the dynamic and multifaceted factors influencing PA behaviors among older people with HIV.

#### Participants and Setting

The target population is sedentary older people with HIV. Inclusion criteria are as follows: (1) age 50 to 80 years, (2) sedentary lifestyle defined as practicing less than 150 minutes of PA per week for the last 6 months [14], (3) known HIV infection, receiving a stable antiretroviral therapy regimen for at least 12 months before enrollment, and (4) owning a smartphone. Participants will be excluded if they have contraindication to PA according to the American College of Sports Medicine [27], including severe arterial hypertension and uncontrolled arrhythmia. Participants will not be excluded based on sex assigned at birth or gender identity. We will recruit ethnically diverse participants from a local community partner that provides HIV or AIDS services to thousands of people in Florida. The sample size in the qualitative interview will be based on thematic saturation. If we do not reach thematic saturation with 15 participants, we will continue the recruitment until we do.

#### Procedure

In-depth interviews will be conducted in person, guided by a predeveloped semistructured interview framework to ensure systematic and comprehensive exploration of participant responses while maintaining focus on the research objectives. Participants will be asked to share their thoughts on the relevance of items derived from the literature review and COM-B framework to their daily PA experiences. They will also be invited to suggest additional factors that may have been overlooked. The semistructured interview guide (detailed in Multimedia Appendix 1) will support this process. Each interview, lasting 30 to 60 minutes, will be audio recorded, transcribed verbatim, and supplemented with the notes of principal investigator. Informed consent will be obtained before participation, and participants will receive a US $20 Amazon gift card as compensation.

#### Data Analysis

For the qualitative data, 2 researchers (SD and XTZ) will independently analyze the transcripts using directed content analysis [28]. The goal is to validate the relevance of the constructs derived from the literature review to the daily PA experiences of older people with HIV and potentially expand the list of constructs. Any coding disagreements will be reviewed, and discrepancies will be resolved through discussion until consensus is reached. A third researcher (YJY) will be involved as needed throughout the process. Through an iterative approach, the final list of construct codes relevant to increased or decreased daily PA will be determined.

### Methods for Aim 2: Implementation of the EMA Study

#### Research Design

**Figure 2:**
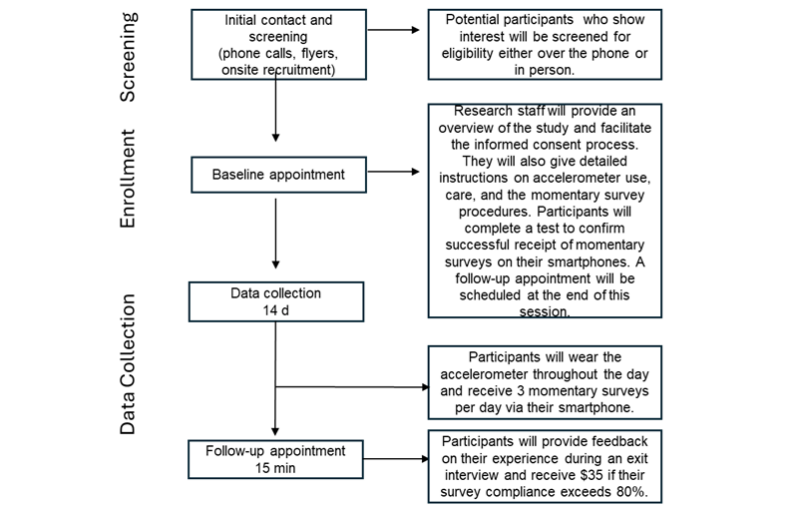
Enrollment and data collection procedures.

This phase of the study involves conducting an EMA study to examine how real-time predictors of PA, identified in aim 1, influence variations in PA levels among sedentary older people with HIV. Participants will be visited twice. During the first visit, they will sign informed consent, provide sociodemographic data (eg, sex, age, educational level, ethnicity, race, and years living with HIV) via an electronic survey and receive instructions for the data collection through EMA surveys. Following this visit, participants will enter a 14-day monitoring period, during which they will complete 3 EMA surveys daily via text message and wear an accelerometer to monitor their PA level. After the 14 days, a second appointment will take place to conduct a brief exit interview to assess any challenges encountered during the EMA study and gather feedback on preferences for future PA interventions. Figure 2 shows a timeline of enrollment and data collection procedures.

#### Participants and Setting

The sample size for the EMA study will be determined through power analysis. All models will use fixed effects, a standard diagonal matrix, and group mean centering of predictors to address clustering of observations within participants. Under these conditions, a sample size of 55 will provide a power of 0.80 to detect moderate effect sizes (β>.40) in multilevel analyses. This calculation assumes up to 5 predictors with moderate multicollinearity (variance inflation factor 2.0), a moderate intraclass correlation coefficient of 0.50, and a significance level (α) of .05. Additionally, to account for potential attrition, a sample size of 70 participants is determined, factoring in a 20% attrition rate.

#### EMA Survey

The COM-B framework serves as a guiding framework for compiling the initial version of the questionnaire, incorporating items previously used in research. These items encompass aspects, such as fatigue, pain, mood, intention, and social support. Subsequently, as part of aim 1, semistructured interviews will be conducted with older sedentary people with HIV. Insights gathered from these interviews will inform further adjustments to the questionnaire, ensuring its relevance and comprehensiveness. Each survey construct will be assessed using a 4-point Likert scale, ensuring the assessment is brief and minimizes participant burden [29].

#### PA Level

To measure participants’ PA levels, all participants will wear a Fitbit wristband on their nondominant wrist for 14 consecutive days during the time-based EMA period. Fitbit wristbands are widely recognized for their reliability and validity in assessing PA, as demonstrated in numerous studies [30]. Participants will be instructed to wear the device throughout the day and night, removing it only for water-based activities. PA measurements will include step count and minutes of MVPA. Fitbit’s proprietary algorithm calculates MVPA minutes (referred to as “active minutes”) by comparing the participant’s current energy expenditure to their resting energy expenditure. When the ratio is ≥3, the algorithm classifies the activity as MVPA [31]. Fitbit data will be retrieved using the manufacturer’s consumer-facing data portal and application programming interface, allowing researchers to download participants’ minute-by-minute data.

#### EMA Survey Schedule, Distribution, and Monitoring

EMA surveys will be delivered via text messages containing embedded web links using the digital health platform of the Florida State University College of Nursing [32]. This platform has been proven effective in supporting digital behavioral interventions by distributing real-time content [33]. EMA survey schedule will begin the day after the baseline visit and follow a fixed-interval schedule with 3 assessments daily (10 AM, 1 PM, and 4 PM) over 14 consecutive days. These times are strategically chosen to capture PA patterns throughout the day (ie, morning, afternoon, and evening) while minimizing recall errors. Building on a previous National Institutes of Health–funded EMA study that explored PA predictors among middle-aged women using a 10-day protocol [34], we extended the time frame to 14 days for this study. Participants will receive a text message at each designated time, with a second reminder sent 30 minutes later if the first goes unanswered. Participants may complete the survey immediately or postpone it for up to 30 minutes, after which the survey will close until the next scheduled time. Participants who complete more than 80% of the surveys during the 14-day EMA study will receive a US $35 Amazon gift card as a token of appreciation.

#### Data Analysis

We will first examine within-person (day-by-day) and between-person (stable over time) variation on each of the PA measures (steps and MVPA minutes) using graphical displays and intraclass correlation coefficients. Then, we will test the effects of within-person survey variables on PA measures via within-person multilevel models using SPSS (version 27; IBM Corp). Our models will use restricted maximum likelihood estimation to generate β coefficients, which compensates for missing data by imputing a distribution within each participant’s scores based on all available data points.

Analyses examined the lagged effects of candidate predictors occurring during a specified time interval (T−1) on PA level (step counts and MVPA minutes) during the subsequent (lagged) time interval (T) within each day of monitoring. For example, it is determined whether fatigue reported at 10 AM predicts subsequent PA reported at 1 PM on that same day. Each predictor’s effect on each of the 2 outcome variables will be tested separately. For each outcome variable, each possible predictor will be tested individually using a minimally restrictive α=.05 level. All variables that pass this screening step will then be combined into a single multivariable model to identify the most parsimonious set of predictors that together account for daily variations in PA among older people with HIV. In the multivariable models, predictors will be entered in block order, and backward elimination will be used to remove redundant predictors within each block and in the final model. Age and gender will be considered as covariates and will be examined as person-level moderators of within-person relations.

### Ethical Considerations

Ethical approval for this study was obtained from Florida State University Institutional Review Board on July 19, 2024 (STUDY00005096). This study’s protocol adheres to all relevant ethical guidelines for research involving human participants, including medical records and patient information.

Informed consent will be obtained from all participants before data collection. During the consent process, participants will receive detailed instructions about this study, including its objectives, procedures, potential benefits, and any associated risks or discomforts. They are also informed of their rights, including the ability to withdraw from this study at any time, to ensure that their participation is completely voluntary. This comprehensive approach is designed to ensure that all participants fully understand this study before providing their consent.

To protect participants’ privacy, all data collected will be anonymized by assigning each participant with a study number to ensure that no personally identifiable information is directly linked to the data. Demographic, personal, and exercise test data will be securely stored in REDCap (Research Electronic Data Capture; Florida State University) databases, which are secure, password-protected databases. Access to these data will be limited to authorized research personnel, and all information obtained during this study will be used for research purposes only. Participants will receive US $35 for their completion of the EMA survey. In addition, participants will receive US $20 for the interview sessions to ensure fairness and transparency in recognition of their time and effort.

## Results

**Figure 3:**
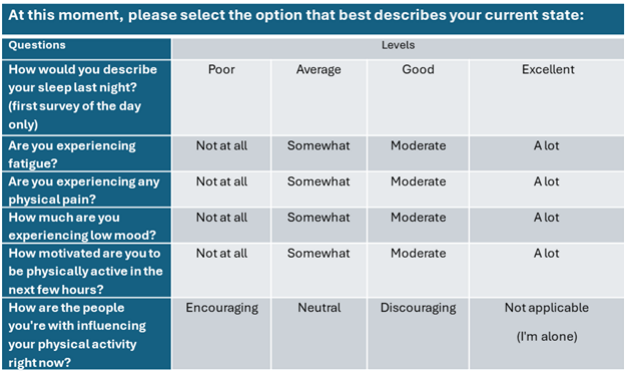
Display of the ecological momentary assessment survey.

To date, 13 eligible participants have completed the qualitative interviews for aim 1. The median age of the participants is 63 (range 57-74) years. Of the 13 participants, 7 identified as African American, 6 identified as White, and the majority identified as male, with 3 identifying as female. Additionally, 6 participants reported not completing a bachelor’s degree, whereas others had attained an associate’s or technical degree. All participants agreed that the listed factors such as pain, fatigue, intention, mood, and social support, are relevant to their PA experience and acknowledged their time-varying nature. For instance, 1 participant remarked, “Pain is always there; some days it’s worse, some days it’s better.” These fluctuations were noted to significantly influence their engagement in PA. Additionally, when asked about other factors they felt were missing from the list, 4 participants identified weather as a contextual factor that plays an important role in their PA experience. A total of 3 participants mentioned that bad sleep was associated with fatigue, suggesting that sleep quality should also be included as a relevant factor. Regarding the EMA survey schedule and distribution, all participants found the proposed schedule (10 AM, 1 PM, and 4 PM) to be acceptable. When shown a prototype of the EMA survey link, participants agreed that it was easy to complete. On the basis of the participants’ input, the EMA survey has been finalized, as shown in Figure 3. To incorporate the weather data identified as relevant, we will document local weather conditions during data collection.

## Discussion

### Anticipated Findings

The aim of this project is to use an EMA approach to capture ecologically valid relations between the personal experiences of older people with HIV and PA as they engage in their normal daily activities. This paper describes our approach in developing this EMA study and highlights existing challenges and future directions.

### Methodological Advantages

First, we adopted a human-centered approach by actively incorporating participants’ feedback into the study design, including the confirmation of relevant factors, such as pain and fatigue, the identification of new factors, such as weather and sleep, and the evaluation of the acceptability of the EMA schedule. This iterative process ensured that the study design is both participant-informed and closely aligned with the needs, preferences, and lived experiences of the target population.

Additionally, the use of EMA surveys represents a significant methodological strength, as it enables the collection of real-time data on factors influencing PA, thereby minimizing recall bias and enhancing data accuracy. This approach also facilitates a deeper understanding of the dynamic and context-dependent influences on PA engagement. By capturing both group-level trends and individual-level variations, EMA surveys offer a comprehensive view of the interpersonal and intrapersonal interplay between personal experiences and contextual factors that shape PA engagement. This dual-level insight is critical for tailoring interventions to address both shared and unique barriers to PA within the target population.

### Methodological Challenges and Decisions

While EMA study design is widely recognized as a robust research method for capturing behaviors and experiences in real-time and naturalistic settings, it does present several limitations, particularly in relation to participant burden and compliance [35]. The repeated prompts required in EMA can be demanding, potentially leading to lower response rates or incomplete data. To mitigate these challenges, we have designed the EMA prompts to be brief, requiring only 2 to 3 minutes to complete, thereby reducing the time burden for participants. Furthermore, participants will have the flexibility to postpone prompts when necessary, allowing them to balance study participation with their daily routines.

To further address compliance concerns, the principal investigator will provide clear, detailed instructions and regular reminders to participants, emphasizing the importance of timely responses. Additionally, an incentive structure has been incorporated to enhance motivation and maintain engagement throughout the study.

Another limitation of this EMA study is its reliance on convenience sampling, which may introduce sampling bias and restrict the generalizability of the findings to broader populations. However, the representativeness of the participants in the aim 1 qualitative studies aligns closely with the demographic characteristics of the national population of older people with HIV, providing a reasonable degree of confidence in the relevance of the findings to this target group. These strategies collectively aim to address the inherent limitations of this EMA study while maximizing the validity and applicability of the study outcomes.

### Significance and Future Directions

Most older people with HIV are sedentary, and increasing PA within this population has remained an elusive goal. Behavioral interventions that rely on “one-size-fits-all” approaches have consistently failed to achieve meaningful increases in PA levels among sedentary older people with HIV [36-38]. To optimize personalization and sustain PA engagement, it is crucial to leverage real-time data to dynamically tailor behavioral support to individuals’ unique, moment-to-moment challenges.

JITAIs offer a promising framework for addressing these challenges. Designed to deliver the right support at the right time, JITAIs adapt to an individual’s changing context and needs [39]. A foundational step in developing a JITAI to promote PA in older people with HIV is understanding the determinants of daily variations in PA levels. While cross-sectional studies have identified between-person correlates of PA, they offer limited insight into how day-to-day experiences and situational factors (within-person determinants) influence PA levels. This study aims to directly address this knowledge gap by using an EMA design that captures both between-person differences and within-person variations in PA. This approach allows a more comprehensive understanding of the dynamic factors influencing PA engagement among this underserved clinical population.

The findings will inform the development of tailored intervention strategies for a JITAI, providing real-time, personalized support to promote PA engagement. This work not only advances the field of precision lifestyle interventions but also addresses critical gaps in promoting healthy aging and improving quality of life for older people with HIV.

## Appendix

Multimedia Appendix 1 Interview guide for exploring physical activity experiences among older people with HIV.

## Abbreviations

COM-B: capability, opportunity, motivation, and behavior
EMA: ecological momentary assessment
JITAI: just-in-time adaptive intervention
MVPA: moderate to vigorous physical activity
PA: physical activity
REDCap: Research Electronic Data Capture

## Data Availability

The datasets generated or analyzed during this study are available from the corresponding author on reasonable request.
