# Supplementary material for: Development of an Ecological Momentary Assessment Study to Identify Real-Time Predictors of Physical Activity Among Older People With HIV: Protocol for a 2-Phase Mixed Methods Study
Source: JMIR Res Protoc. 2025 Dec 9;14:e81238. doi: 10.2196/81238 (PMC12728394; doi:10.2196/81238)
Supplement: Multimedia Appendix 1 [file resprot_v14i1e81238_app1.docx]

**Interview Guide**

Thank you for participating in this interview. The purpose of this discussion is to explore which factors influence your engagement in daily physical activities. Your input will help us better understand your physical activity experience and inform strategies to support your physical activity.

1. How would you describe your current level of physical activity on a typical day?
2. whether your physical activity level varies day by day?
3. What are the factors that you think lead to the fluctuations of physical activity?
4. Here is a list of factors that potentially lead to the fluctuations of daily physical activity, do you agree with this list?

| Questions | Levels | | | |
| --- | --- | --- | --- | --- |
| Are you experiencing **fatigue**? | Not at all | Somewhat | Moderate | A lot |
| How would you describe your **sleep** last night? | Poor | Average | Good | Excellent |
| Are you experiencing any **physical pain?** | Not at all | Somewhat | Moderate | A lot |
| How much are you experiencing **low mood?** | Not at all | Somewhat | Moderate | A lot |
| How **motivated** are you to be physically active in the next few hours | Not at all | Somewhat | Moderate | A lot |
| How are the **people you're with** influencing your physical activity right now? | Encouraging | Neutral | Discouraging | Not applicable (I'm alone) |

1. What other factors do you think is missing from this list?
2. Can you share any strategies or techniques you use to overcome barriers and stay motivated to be physically active?
3. Based on your experiences, what recommendations would you offer to healthcare providers to better support physical activity?
4. Is there anything else you would like to share or discuss related to physical activity and healthy aging?
